# Supplementary material for: AggreBots: configuring CiliaBots through guided, modular tissue aggregation
Source: bioRxiv. 2025 Feb 27:2025.02.22.639695. Preprint. [Version 1] doi: 10.1101/2025.02.22.639695 (PMC11888266; doi:10.1101/2025.02.22.639695)
Supplement: Supplement 1 — Supplementary Fig. S1. Inability of mature CiliaBots to aggregate. Supplementary Fig. S2. Aggregation success rate as function of CBB age. Supplementary Fig. S3. Schematic depiction of process behind characterization of CiliaBot motility. [file media-1.docx]

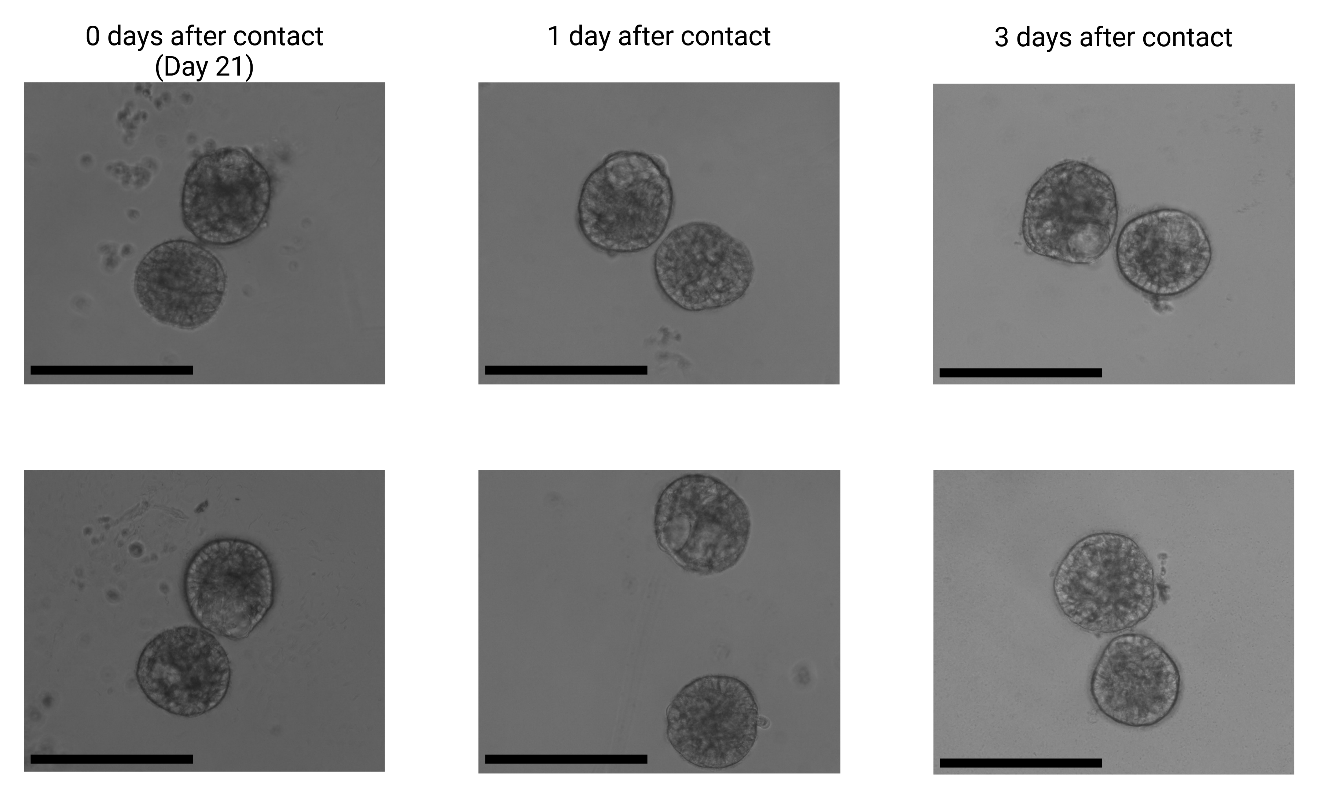


**Supplementary Fig. S1.** **Inability of mature CiliaBots to aggregate.** CiliaBots were brought into contact with one another on Day 21. Images were subsequently taken 1 and 3 days following initial contact, showcasing that mature CiliaBots appear to have no tendency to aggregate.


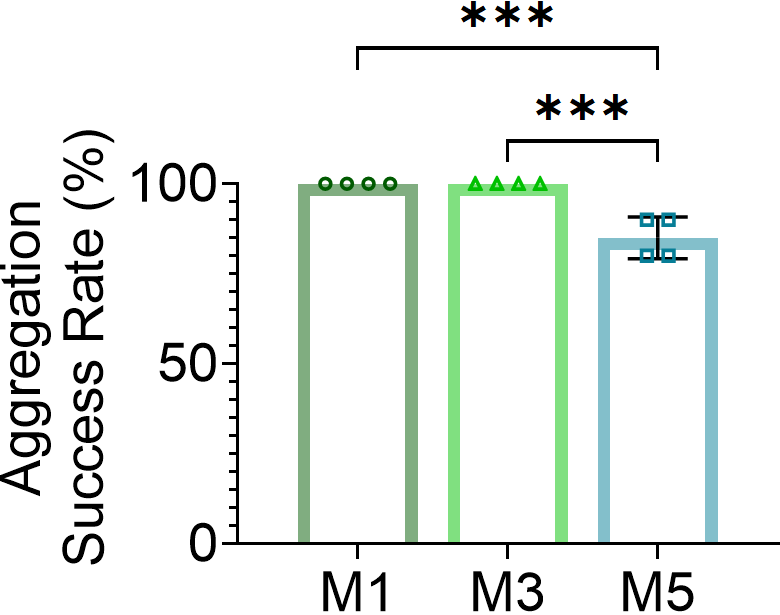


**Supplementary Fig. S2.** **Aggregation success rate as function of CBB age.** Aggregation success rates of each CBB age pre-contact group (M1, M3, M5). Each data point represents a separate experimental batch of CBBs.


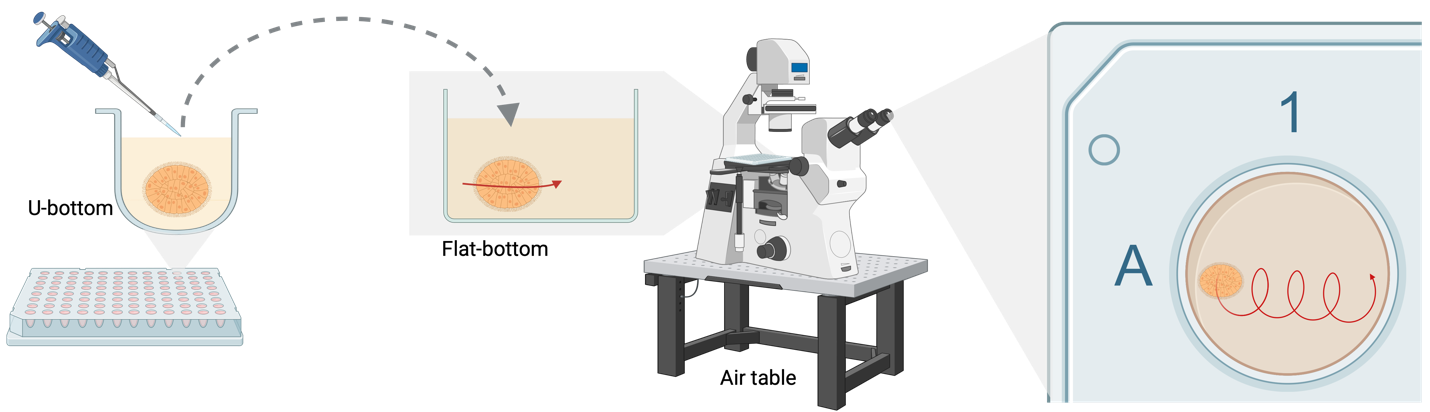


**Supplementary Fig. S3.** Schematic depiction of process behind characterization of CiliaBot motility.

**Supplementary Movie S1.** Exterior cilia agitate CiliaBots, preventing stable contact and aggregation.

**Supplementary Movie S2.** 30X Speed video depicting locomotion of a CBB_2_ AggreBot, alongside visualization of the traced loop-de-loop via the “spine-and-vertebrae” method.

**Supplementary Movie S3.** Locomotion of a CBB_3_ AggreBot.

**Supplementary Movie S4.** Locomotion of a CBB_4_ AggreBot.

**Supplementary Movie S5.** Locomotion of a ^H^CBB UniBot.

**Supplementary Movie S6.** Lack of motility from a ^PCD^CBB UniBot.

**Supplementary Movie S7.** Cilia activity of a collagen-embedded ^H^CBB_1_^PCD^CBB_1_ AggreBot.

**Supplementary Movie S8.** Cilia activity of a collagen-embedded ^H^CBB_2_^PCD^CBB_1_ AggreBot.

**Supplementary Movie S9.** Cilia activity of a collagen-embedded ^H^CBB_1_^PCD^CBB_2_ AggreBot.

**Supplementary Movie S10.** Locomotion of a ^H^CBB_2_ AggreBot.

**Supplementary Movie S11.** Locomotion of a ^H^CBB_1_^PCD^CBB_1_ AggreBot, showcasing the decreased translational speed and increased path curvature brought about by the incorporation of ^PCD^CBBs, with ^PCD^CBB marked with red “vertebrae”.

**Supplementary Movie S12.** Locomotion of a ^H^CBB_3_ AggreBot.

**Supplementary Movie S13.** Locomotion of a ^H^CBB_1_^PCD^CBB_2_ AggreBot.

**Supplementary Movie S14.** Locomotion of a ^H^CBB_4_ AggreBot.

**Supplementary Movie S15.** Locomotion of a chiral ^H^CBB_2_^PCD^CBB_2_ AggreBot.
